# MicroRNA-26a inhibits osteosarcoma cell proliferation by targeting IGF-1

**DOI:** 10.1038/boneres.2015.33

**Published:** 2015-12-22

**Authors:** Xinyu Tan, Shicai Fan, Wen Wu, Yin Zhang

**Affiliations:** 1 Department of Orthopaedics, The Third Affiliated Hospital of Southern Medical University, Guangzhou, China

## Abstract

There are still controversies about the roles of microRNA-26a (miR-26a) in human malignancies, as it is a tumor suppressor in breast cancer, gastric cancer, and hepatocellular carcinoma, but is an oncogene in glioma and cholangiocarcinoma. Until now, the function of miR-26a in osteosarcoma remains largely elusive. Here, we found that miR-26a was downregualted in osteosarcoma tissues. Using *in vitro* and *in vivo* assays, we confirmed that miR-26a could inhibit the abilities of *in vitro* proliferation and suppress *in vivo* tumor growth in mouse model. Furthermore, we identified insulin-like growth factor 1 (IGF-1) as a novel and direct target of miR-26a and revealed that miR-26a exerted its tumor-suppressor function, at least in part, by inhibiting IGF-1 expression. These findings contribute to our understanding of the functions of miR-26a in osteosarcoma.

## Introduction

Osteosarcoma is the most common primary malignancy, and it arises primarily in the metaphysis of the long bones in adolescents and young adults.^1,2^ It leads to many deaths because of its rapid proliferation and metastasis.^3^ Recent studies have shown that microRNAs (miRNAs) are involved in various cancer-related processes.^4^ miRNAs are a group of endogenously expressed, non-coding small RNAs (20–25 nucleotides in length). miRNAs negatively regulate the expression of target messenger RNAs (mRNAs) by suppressing translation or decreasing the stability of mRNAs.^5^ It has been found that miRNAs play crucial roles in various biological processes, including development, differentiation, apoptosis, and cell proliferation.^6^ An increasing number of studies have demonstrated that miRNAs can function as oncogenes or tumor suppressors, and they are often dysregulated in tumors.^7–10^

There are still controversies about the roles of miR-26a in human malignancies, as it is a tumor suppressor in breast cancer,^11^ gastric cancer,^12^ and hepatocellular carcinoma,^13,14^ but is an oncogene in glioma^14^ and cholangiocarcinoma.^15^ Although miR-26a was found to be downregulated in osteosarcoma previously,^16^ its biological function and precise mechanism in osteosarcoma remain largely elusive.

In this study, we confirmed the downregulation of miR-26a in osteosarcoma tissues. Using both gain- and loss-of-function analyses, we further revealed that miR-26a suppressed osteosarcoma cell proliferation *in vitro* and *in vivo*. Moreover, we revealed that insulin-like growth factor 1 (IGF-1) is a target of miR-26a, and miR-26a exerted its tumor-suppressor function, at least in part, by inhibiting IGF-1 expression.

## Materials and methods

### Cell lines and culture

Osteosarcoma cell lines MG-63 and U2OS were purchased from the Cell Resource Center of the Institute of Basic Medical Sciences at the Chinese Academy of Medical Sciences. These cells were cultured in Roswell Park Memorial Institute 1640 medium (Gibco, Waltham, MA, USA) supplemented with 10% fetal bovine serum (Gibco) at 37 °C with 5% CO_2_. Human embryonic kidney 293 (HEK293) cells were maintained in Dulbecco's modified Eagle's medium (Gibco) supplemented with 10% fetal bovine serum (Gibco).

### Clinical specimens

Tumor tissues and adjacent nontumor normal tissues were collected from routine therapeutic surgery at our department after obtaining informed consent in accordance with a protocol approved by the Ethics Committee of Southern Medical University (Guangzhou, China).

### Quantitative RT-PCR analysis

The total RNAs were extracted from cells with TRIZOL reagent (Invitrogen, Carlsbad, CA, USA). For the detection of miR-26a, RT and PCR reactions were performed by means of qSYBR-green-containing PCR kit (GeneCopoeia, Rockville, MD, USA), and U6 snRNA was used as an endogenous control for miRNA detection. For IGF-1 mRNA, cDNA was synthesized from 1 μg of total RNA by means of Reverse Reaction kit according to the manufacturer’s instructions (Promega, Madison, WI, USA). Human glyceraldehyde-3-phosphate dehydrogenase (GAPDH) was amplified in parallel as an internal control. The expression of each gene was quantified by measuring Ct values, and normalized using the 2^−ΔΔCt^ method relative to U6 snRNA or GAPDH.

### *In vitro* cell proliferation assays

Transfected cells were plated on 12-well plates at the desired cell concentrations and cell counts were estimated by trypsinizing the cells and performing analysis using a Coulter Counter (Beckman Coulter, Fullerton, CA, USA) at the indicated time points in triplicate. Meanwhile, transfected cells were plated on 96-well and the cell proliferation was measured by MTS-formazan reduction (Promega, Madison, WI, USA) by absorbance at 450 nm.

### Luciferase reporter assay

IGF-1 3′ untranslated region (UTR) was amplified from human blood genomic DNA and then was cloned into pMir-Report (Abcam, Cambridge, MA, USA). Yielding mutant constructs, mutations were introduced in potential miR-26a binding sites using the QuikChange site-directed Mutagenesis Kit (Stratagene, La Jolla, CA, USA). One microgram of the wild type or mutant UTR of IGF-1 were cotransfected either with 50 nmol⋅L^−1^ of miR-26a mimics or negative control (NC) into HEK293 cells using Lipofectamine 2000 (Invitrogen). Cells were harvested 48 h after transfection and assayed using the Dual Luciferase Reporter Assay System (Promega).

### Western blot analysis

Proteins were separated by 10% sodium dodecyl sulfate-polyacrylamide gel electrophoresis and then transferred to polyvinylidene fluoride membranes (Amersham, Buckinghamshire, UK). The membranes were incubated overnight at 4 °C with anti-IGF-1 antibody (Abcam) and anti-GAPDH (Sigma-Aldrich Corp., St. Louis, MO, USA) antibody followed by horseradish peroxidase -linked secondary antibodies.

### Mouse xenograft model

MG-63 cells infected either with miR-26a, anti-miR-26a, or NC lentiviruses (Genecopies) were inoculated subcutaneously into the dorsal flanks of nude mice (five in each group). Tumor volumes were monitored every 5 days. After 30 days, the mice were killed, necropsies were performed, and the tumors were weighed. All mouse experiments were performed according to the Institutional Animal Care and Use Committee procedures and guidelines.

### Statistical analysis

Statistical analyses were performed using SPSS 16.0. Data are presented as the mean ± standard deviation. The difference between groups was analyzed using a Student’s *t-*test when comparing only two groups or one-way analysis of variance when comparing more than two groups. *P* < 0.05 was considered statistically significant.

## Results

### miR-26a is downregulated in osteosarcoma

**Figure 1:**
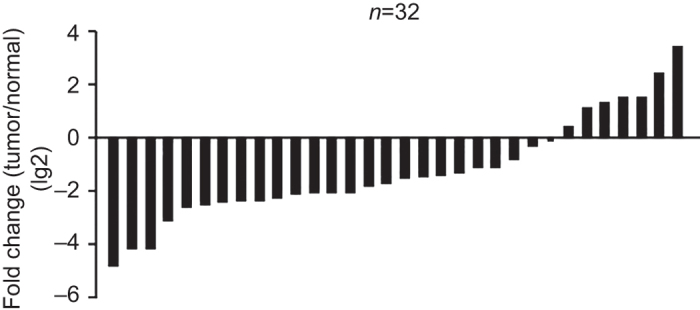
The expression of miR-26a in human osteosarcoma tissues. miR-26a was detected in 32 osteosarcoma patients by qRT-PCR. Data are presented as the lg2 fold-change in osteosarcoma tissues relative to the adjacent normal tissues.

To investigate the role of miR-26a in human osteosarcoma, we first examined miR-26a expression in *n* = 32 pairs of osteosarcoma tissues and pair-matched adjacent noncancerous tissues using quantitative qRT-PCR. Consistent with the previous report,^16^ miR-26a was significantly downregulated in osteosarcoma tissues compared to the paired bone tissues. Among the 32 patients with osteosarcoma, approximately 79% (22 of 32 patients) of tumors revealed a more than twofold reduction in miR-26a levels, with a 5.76-fold reduction relative to adjacent normal tissues (Figure 1). These results suggest that the miR-26a might play a critical role in the osteosarcoma initiation.

### miR-26a suppresses osteosarcoma cell proliferation

**Figure 2:**
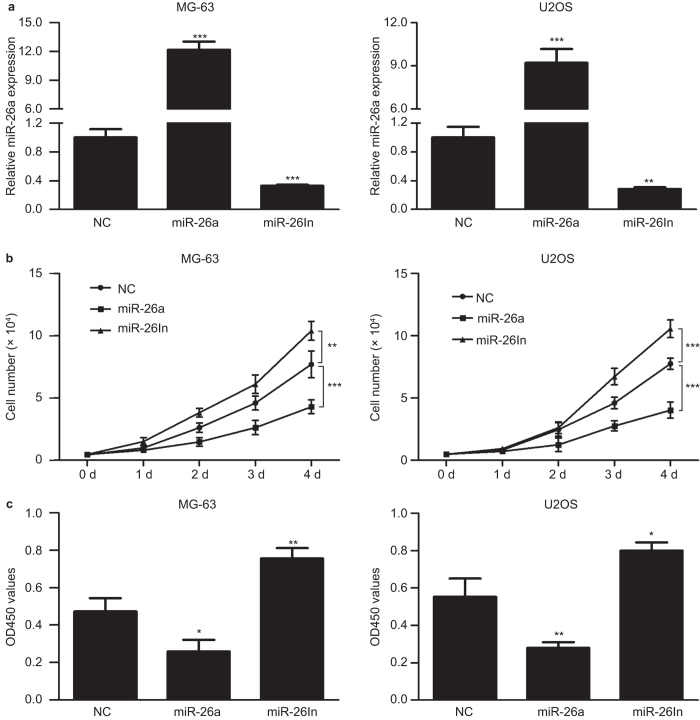
miR-26a inhibits osteosarcoma cell proliferation. (**a**) Examination of miR-26a expression in MG-63 and U2OS cells transfected with NCs, miR-26a mimics or inhibitors. (**b**) Growth assays were performed by cell counting. MG-63 and U2OS cells were transfected with NC, miR-26a mimics, or inhibitors. (**c**) The cell proliferative potential was determined in MG-63 and U2OS cells. A450 absorption was assayed after transfection for 72 h. Data are presented as mean ± s.d. from at least three independent experiments. *P < 0.05; **P < 0.01; ***P < 0.01.

To evaluate the biological significance of miR-26a in the development of osteosarcoma, we transfected with miR-26a mimic or inhibitor into MG-63 and U2OS cells (Figure 2a), and examined cell proliferation using direct cell counting and MTS assays. Overexpression of miR-26a inhibited cell proliferation, whereas inhibition of miR-26a promoted cell growth (Figure 2b and c).

### miR-26a suppresses tumor growth in mouse xenografts

**Figure 3:**
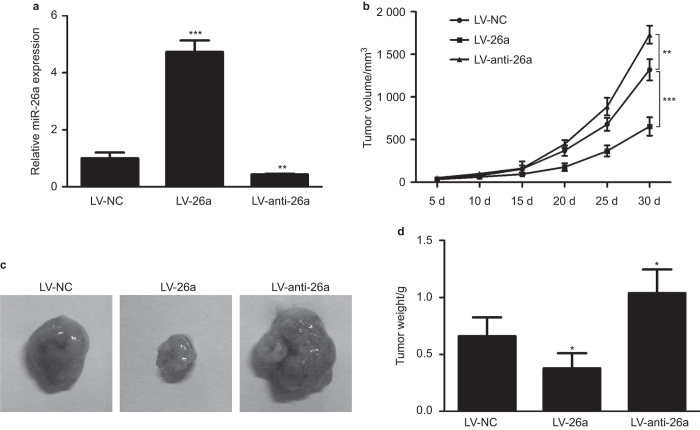
miR-26a attenuated tumor growth in mouse xenograft models. (**a**) Examination of miR-26a expression in MG-63 cells stably infected with NC, miR-26a, or anti-miR-26a lentivirus. (**b–d**) Tumor growth in mouse xenograft models. MG-63 cells infected with NC, miR-26a, or anti-miR-26a lentivirus were injected subcutaneously into nude mice. Tumor size was measured every 5 days. After 30 days, the mice were killed, necropsies were performed, and tumors were weighed. Data are presented as mean ± s.d. *P < 0.05; **P < 0.01; ***P < 0.01.

To investigate the *in vivo* effects of miR-26a on osteosarcoma tumorigenesis, we stably overexpressed and knocked down miR-26a in MG-63 cells by lentivirus. The efficiency was confirmed through RT-PCR (Figure 3a). Next, nude mice transplanted with MG-63 cells infected either with miR-26a, anti-miR-26a, or NC. We found that the overexpression of miR-26a in MG-63 cells significantly suppressed tumor growth in nude mice (Figure 3b–d). In contrast, knockdown of miR-26a in MG-63 cells was found to promote tumor growth in mice Figure 3b, c, and d). These results indicate that miR-26a may repress osteosarcoma tumorigenesis.

### miR-26a inhibits cell proliferation by targeting IGF-1 in osteosarcoma cells

**Figure 4:**
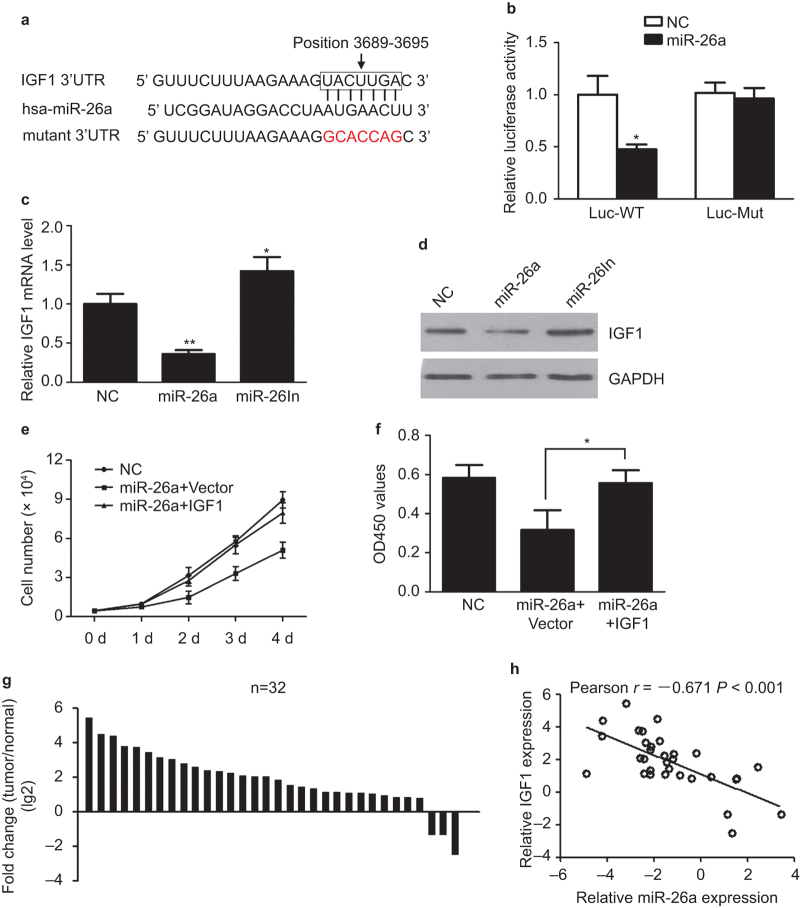
IGF-1 is a direct target of miR-26a. (**a**) IGF-1 3’-UTR contains one predicted miR-26a binding site. The mutagenesis nucleotides are indicated in red. (**b**) Dual luciferase reporter assay. HEK293 cells were transfected with wild-type 3’-UTR-reporter or mutant (Mut) constructs together with miR-26a mimics or NCs. Relative firefly luciferase expression was normalized to Renilla luciferase. (**c** and **d**) qRT-PCR and Western blot to measure IGF-1 mRNA and protein level in MG-63 cells transfected with NC, miR-26a mimics, or inhibitors. (**e** and **f**) IGF-1 rescues the suppressive roles of miR-26a in GC cell proliferation. MG-63 cells expressing miR-26a mimics or NC were transfect with or without IGF1 plasmids. Cell proliferation analysis was performed by cell counting (**e**) and MTS assay (**f**). (**g**) The expression of IGF-1 in 32 pairs of osteosarcoma samples and matched adjacent noncancerous tissues by qRT-PCR. Data are presented as the lg2 fold-change in osteosarcoma tissues relative to the adjacent normal tissues. (**h**) The correlation between miR-26a expression and IGF-1 expression in osteosarcoma tissues. Data are presented as mean ± s.d. from at least three independent experiments. *P < 0.05; **P < 0.01.

Using *in silico* prediction programs, we identified IGF-1 as a potential target for miR-26a. The 3’-UTR of IGF-1 mRNA (position 3689–3695 of IGF-1 3’-UTR) harbored sequences complementary to the miR-26a seed sequence (Figure 4a) and the seed-recognizing region is conserved across species. To verify whether IGF-1 is a direct target of miR-26a, we cloned the wild-type 3’-UTR or the mutant (lacking the 7-bp seed sequence) into a luciferase reporter vector. When we cotransfected HEK293 cells with the cloned 3’-UTR and miR-26a mimics, we observed a consistent reduction in luciferase activity for 3’-UTR by miR-26a (Figure 4b). Conversely, cotransfection of miR-26a mimics with the mutated form of the 3’-UTR resulted in no significant change in luciferase activity (Figure 4b), suggesting miR-26a directly targets the IGF-1 3’-UTR. In agreement, miR-26a overexpression significantly reduced both mRNA and protein expression for IGF-1 in MG-63 cells (Figure 4c and d). Furthermore, miR-26a inhibitors transfection increased its mRNA and protein levels, further indicating that IGF-1 is a target of miR-26a in osteosarcoma cells.

The above results prompted us to examine whether miR-26a suppresses osteosarcoma growth by inhibiting IGF-1 expression. For this purpose, we transfected plasmid pReceiver containing IGF-1 or empty plasmid in MG-63 cells transfected with miR26a to recover IGF-I expression. IGF1 was overexpressed in miR-26a-transfected MG-63 cells. In miR-26a-expressing cells, overexpression of IGF-1 rescued growth defects of miR-26a (Figure 4e and f).

Finally, we examined IGF1 expression in *n*=32 pairs of osteosarcoma and matched adjacent tissues by qRT-PCR. The result showed that IGF-1 expression was increased in osteosarcoma relative to the paired bone tissues (Figure 4g). There was an inverse correlation between miR-26a expression and IGF1 expression in osteosarcoma tissues (Figure 4f)

## Discussion

Recent studies have revealed a critical role for miRNAs in tumor initiation and progression, including in osteosarcoma.^17–19^ In the present study, we determined that the level of miR-26a expression was significantly lower in osteosarcomas than that in adjacent nontumor tissue. Using *in vitro* and *in vivo* assays, we identify the tumor suppressor function of miR-26a in osteosarcoma. By upregulating and downregulating miR-26a in osteosarcoma cells, we confirmed that miR-26a could inhibit the abilities of *in vitro* proliferation and suppress *in vivo* tumor growth. Furthermore, we also identified IGF-1 as a novel and direct target of miR-26a. Our findings suggest that miR-26a has a suppressor role in osteosarcoma tumorigenesis.

Several previous studies support our results. For example, miR-26a is decreased in hepatocellular carcinoma (HCC) and could suppress tumor angiogenesis of HCC through hepatocyte growth factor -cMet signalling.^20^ Breast cancer also exhibits decreased expression of miR-26a and overexpression of this miRNA results in inhibition of tumor growth and metastasis.^11^ However, others indicate that it exhibits oncogenic properties in glioma^14^ and cholangiocarcinoma.^15^ For example, miR-26a is overexpressed in cholangiocarcinoma and promotes cholangiocarcinoma growth by activating B-catenin.^15^ These controversial results suggested that the role of miR-26a was possibly tumor-specific and highly dependent on its targets in different cancer cells. Various studies have shown that PTEN,^21^ EZH2,^22,23^ SMAD1,^24^ CDK6, and cyclin E1^25^ are potential downstream target genes of miR-26. In this study, we found that IGF-1 serves as a downstream mediator of tumor suppressor function in osteosarcoma.

It is well known that IGF-1 plays important roles in cell proliferation, motility, and metastasis.^26,27^ Amplified IGF-1/IGF-1R signaling is not only associated with development of tumors, but also contributes to tumor cell survival, invasion, metastasis, and resistance to chemotherapeutic drugs.^27,28^ In our studies, we confirmed that IGF-1 was a direct target of miR-26a in osteosarcoma cells. To determine whether miR-26a suppresses osteosarcoma cell proliferation through targeting IGF-1, we found that IGF-1 overexpression could rescue growth inhibition of miR-26a. Moreover, there was an inverse correlation between miR-26a expression and IGF-1 expression in osteosarcoma tissues. These results suggest that miR-26a inhibits osteosarcoma cell proliferation partly by targeting IGF1.

Taken together, the current study provided novel evidence that miR-26a is significantly downregulated in osteosarcoma clinical specimens and appears to function as a tumor suppressor in osteosarcoma through the regulation of IGF-1 expression and cell proliferation. These results may help us understand the molecular mechanism of osteosarcoma tumorigenesis, and provide us with a strong rationale to further investigate miR-26a as a potential biomarker and therapeutic target for osteosarcoma.
